# Compensatory growth following transient intraguild predation risk in predatory mites

**DOI:** 10.1111/oik.01687

**Published:** 2015-05-01

**Authors:** Andreas Walzer, Natalia Lepp, Peter Schausberger

**Affiliations:** Div. of Plant Protection, Dept of Crop Sciences, Univ. of Natural Resources and Life Sciences, Peter Jordanstrasse 82, AT-1190 Vienna, Austria.; Div. of Plant Protection and Quarantine, Dept of Agro-technologies, Soil Sciences and Ecology, Saint-Petersburg State Agrarian Univ., Petersburg road 2, RU-196601 Saint Petersburg, Russia.; Div. of Plant Protection, Dept of Crop Sciences, Univ. of Natural Resources and Life Sciences, Peter Jordanstrasse 82, AT-1190 Vienna, Austria.

## Abstract

Compensatory or catch-up growth following growth impairment caused by transient environmental stress, due to adverse abiotic factors or food, is widespread in animals. Such growth strategies commonly balance retarded development and reduced growth. They depend on the type of stressor but are unknown for predation risk, a prime selective force shaping life history. Anti-predator behaviours by immature prey typically come at the cost of reduced growth rates with potential negative consequences on age and size at maturity. Here, we investigated the hypothesis that transient intraguild predation (IGP) risk induces compensatory or catch-up growth in the plant-inhabiting predatory mite *Phytoseiulus persimilis*. Immature *P. persimilis* were exposed in the larval stage to no, low or high IGP risk, and kept under benign conditions in the next developmental stage, the protonymph. High but not low IGP risk prolonged development of *P. persimilis* larvae, which was compensated in the protonymphal stage by increased foraging activity and accelerated development, resulting in optimal age and size at maturity. Our study provides the first experimental evidence that prey may balance developmental costs accruing from anti-predator behaviour by compensatory growth.

Environmental stress during development commonly interferes with growth, prolonging developmental time and possibly reducing body size at maturity (Metcalfe and Monaghan 2001, Dmitriew 2011, Hector and Nakagawa 2012). However, if stress is only transient and followed by benign conditions the detrimental fitness effects of limited growth on age and size at maturity may be compensated for by catch-up or compensatory growth (Hector and Nakagawa 2012). In catch-up growth, growth rates are optimized to reach optimum adult body size, which comes at the expense of prolonged developmental time. In compensatory growth, growth rates are maximized to reach optimum age and size at maturity without prolonging developmental time (Hector and Nakagawa 2012). Both growth strategies usually have costs paid later in life (Hector and Nakagawa 2012) but are considered adaptive, when the costs associated with higher than optimal growth rates and/or longer than optimal developmental time are lower than the benefits arising from reaching adulthood at optimal age and size (Dmitriew 2011). Common major benefits of optimal age at maturity as compared to prolonged development are higher survival probability during the sensitive developmental phase, earlier mating opportunities and earlier access to food and other resources. Common major benefits of optimal body size at maturity as compared to small size are superiority in food competition and mate choice linked to higher lifetime reproductive success (Peters 1983, Clutton-Brock 1988, Roff 1992, Stearns 1992, Andersson 1994).

Catch-up and compensatory growth strategies following transient stress during development are well known for numerous animals, incl. mammals, birds, fishes, amphibians and arthropods (Wilson and Osbourn 1960, Ashworth and Millward 1986, Dmitriew 2011, Hector and Nakagawa 2012). In most documented cases, the stressors triggering compensatory or catch-up growth were unfavourable abiotic conditions such as low temperature, food limitation or exposure to toxic substances (Dmitriew 2011). Strikingly, evidence for catch-up or compensatory growth following predation risk, one of the prime selective forces shaping life history, is lacking although numerous studies indicate that predation risk may lower the growth rates of prey (Lima and Dill 1990, Van Buskirk and Yurewicz 1998, Benard 2004). The only two studies experimentally assessing growth modulation following a period of predation risk stem from tadpoles. Tadpoles exposed to short-term predation risk followed by a period of favourable conditions were not able to compensate body size reduction induced by predation risk (Altwegg and Reyer 2003, Capellan and Nicieza 2007).

Here we assessed growth and development of intraguild (IG) prey during and after exposure to transient intraguild predation (IGP) risk. IGP, the killing of heterospecific food competitors, is a widespread predator – predator interaction (Polis et al. 1989, Arim and Marquet 2004). A common feature of IG prey–predator interactions is size-selective predation in dependence of the ontogenetic stage and functional role of the guild members. Large developmentally advanced juvenile or adult IG predators preferentially prey on young small juvenile IG prey (Polis et al. 1989). Consequently, juvenile guild members are often exposed to high IGP risk early in life followed by a period of low or negligible risk. This shift in IGP risk correlates with shifting growth conditions of juvenile guild members because effective anti-predator responses are usually traded off against foraging, decreasing the growth rates of prey (Lima and Dill 1990, Abrams 1991, Werner and Anholt 1993, Benard 2004). Decreased growth rates, in turn, commonly result in costly deviations from optimum age and size at maturity (Roff 1992, Stearns 1992). Thus, selection should favour guild members that are able to compensate for a bad start in life by growth recovery under more favourable conditions later in life (Metcalfe and Monaghan 2001, Ali et al. 2003, Hector and Nakagawa 2012).

We tested this hypothesis in the plant-inhabiting predatory mite *Phytoseiulus persimilis*. In the Mediterranean region, *P. persimilis* forms naturally occurring predatory mite guilds together with *Neoseiulus californicus* and *Amblyseius andersoni* (Acari: Phytoseiidae). They share the two-spotted spider mite *Tetranychus urticae* as extraguild prey (Acari: Tretranychidae) (Walzer and Schausberger 2011a). The six-legged and little mobile larvae of *P. persimilis* (Schausberger and Croft 1999) are the most vulnerable IG prey individuals within this guild, with adult *A. andersoni* and *N. californicus* females being high and low risk IG predators, respectively (Walzer and Schausberger 2011a). Although *P. persimilis* larvae do not feed (Schausberger and Croft 1999), growth based on maternally provided internal reserves is evident. It is true for all phytoseiid mites that the life-stage following the larva, the protonymph, has a larger body size and additional, novel structures such as a 4th pair of legs and more setae (Helle and Sabelis 1985, Krantz and Walter 2009). *Phytoseiulus persimilis* larvae are able to distinguish between high and low risk IG predators resulting in threat-sensitive shifts in velocity, distance moved and spatial predator avoidance (Walzer and Schausberger 2013a, b). The fitness tradeoffs of these energy-binding anti-IG predator responses may be retarded development, increasing juvenile mortality risk and postponing mating and reproduction, or smaller body size at maturity (Stearns 1992). The eight-legged stages following the larva, i.e. proto- and deutonymph, are much less endangered to fall victim to IG predators but are themselves often predators of heterospecific larvae (Walzer and Schausberger 1999). Accordingly, regarding the IGP risk the developmental phase of *P. persimilis* can be divided in a stressful larval phase with high IGP risk and suboptimal growth conditions and the subsequent nymphal phase with low IGP risk and thus more favourable growth conditions.

Using a transient IGP scenario, we investigated the effects of graded IGP risk on development and anti-IG predator behaviour of *P. persimilis* larvae, and their subsequent nymphal development under benign conditions, i.e. in physical absence of an IG predator. We hypothesized that short-term exposure to IGP risk has negative consequences on larval growth, which should then be counter-balanced by compensatory or catch-up growth of nymphs because prolonged juvenile development and small body size at maturity are detrimental to fitness (Sabelis 1985, Walzer and Schausberger 2013c, 2014). Since the non-feeding IG prey larvae respond in a threat-sensitive manner to predation risk, we expected that presence of the low and high risk IG predators differently affects larval growth and subsequent growth responses of nymphs under benign conditions.

## Material and methods

### Species origin and rearing

About 20 specimens of *Phytoseiulus persimilis*, *Neoseiulus californicus* and *Amblyseius andersoni* were collected from herbs and apple trees in Trapani, Sicily, in 2007, in order to found laboratory-reared populations. In the laboratory, each species was separately reared on arenas consisting of plastic tiles resting on water-saturated foam cubes in plastic boxes half-filled with water (Walzer and Schausberger 2011a). The predators were fed in 2 to 3 day intervals with their extraguild prey, the spider mite *T. urticae*, reared on whole common bean *Phaseolus vulgaris*. All three predator species were reared in the laboratory for ~3 years before the experiments took place. To obtain similarly aged *P. persimilis* eggs, giving rise to IG prey larvae used in experiments, gravid females were transferred from the rearing units to detached bean leaves and provided with surplus spider mites. After 2 h, the females were removed whereas their eggs were left on the arena until hatching. All rearing and experimental units were kept in a climate chambers at 25 ± 1 °C, 70 ± 5% RH and 16:8 h L:D photoperiod.

### Experimental units

Each experimental unit consisted of an arena (30 × 30 mm) on a single detached bean leaf placed upside down on a water-saturated foam cube in a plastic box half-filled with water. The leaf arena was delimited by strips of moist tissue paper preventing the mites from escaping. To obtain a predefined extraguild prey density of 20 juveniles and 40 eggs, two ovipositing spider mite females were placed on each experimental unit for 24 h and then removed. After another 72 h four ovipositing spider mite females were placed on each experimental unit for 24 h and then removed. Subsequently, the ratio between juvenile spider mites hatched from old eggs and newly deposited eggs was adjusted to 20 juveniles and 40 eggs per arena.

To simulate transient, graded IGP risk and stressful growth conditions, respectively, for the *P. persimilis* larvae, followed by a period of negligible risk and optimal growth conditions for the nymphs, single IG prey larvae were subjected to one of three treatments on leaf arenas: 1) only extraguild prey (control; n = 39); 2) extraguild prey and a low risk IG predator female (*N. californicus*; n = 37); 3) extraguild prey and a high risk IG predator female (*A. andersoni*; n = 46). Gravid IG predator females were randomly chosen from the rearing units and housed singly in closed acrylic cages (Schausberger 1997) for 12 h before use in experiments to increase their IGP propensity. To start the experiment a single 48 h old IG prey egg of *P. persimilis* was placed on the leaf arena with or without an IG predator female. After hatching, which usually occurs within 3 h after placing the 48h old egg on the arena (Walzer and Schausberger 1999), survival and activity (moving/stationary) of IG prey larvae, activity of IG predators and position of both IG prey and IG predator were observed every 3 h throughout the larval stage, until molting to the protonymph, lasting approximately 12 to 15 h at 25 °C (Schausberger and Croft 1999). Position of IG prey and IG predator were marked in paper sketches of the leaf arenas to determine their inter-individual distances. After IG prey had reached the protonymphal stage the IG predator female was removed and the extraguild prey density was replenished to 20 juveniles and 40 eggs. The developmental progress of the nymphs was observed twice daily in 8 and 16 h intervals until IG prey reached adulthood or died on the leaf. Subsequently, the number of intact spider mite eggs and juveniles was counted and the sex of IG prey determined. For body size measurements, each adult *P. persimilis* individual was mounted in a drop of Hoyer’s medium (Krantz and Walter 2009) and then dried at room conditions for two days. Dorsal shield length, which is an appropriate indicator of phytoseiid body size (Croft et al. 1999), was measured under the microscope at 200 × magnification.

### Statistical analyses

All statistical analyses were carried out using SPSS ver. 18.0.1 and the data are available as supporting information (Supplementary material Appendix 1). Separate generalized linear models (GLM) were used to analyse 1) the influence of graded IGP risk (no, low or high) on survival (yes/no), mean activity (proportion of time moving), mean distance to the IG predator (used as an indicator of spatial IG predator avoidance) and developmental time of IG prey larvae, 2) the difference in IG predator (*N. californicus*, *A. andersoni*) activity, 3) and the influence of graded IGP risk (no, low or high) on total developmental time (from larva to adulthood), sex-ratio and sex-specific body size at maturity. Separate generalized estimating equations (GEEs) were used to analyse the influence of graded IGP risk (no, low or high) during the larval stage and of nymphal stage (used as within-subject variable with autocorrelation structure between the proto- and deuto-nymphal stage) on consumption of spider mite eggs and juveniles, mean activity and developmental times of nymphs. We used normal distribution and identity link function for all parameters except survival and sex-ratio where we used binomial distribution and logit link function. All proportional data (activity of IG prey larvae, proto- and deutonymphs and predators) were arcsine square-root transformed before analysis. To detail differences within IGP risk levels (no, low or high), the estimated marginal means were compared between treatment pairs by Fisher’s least significant difference (LSD) tests.

## Results

### Larval IG prey responses to graded IGP risk

**Figure 1:**
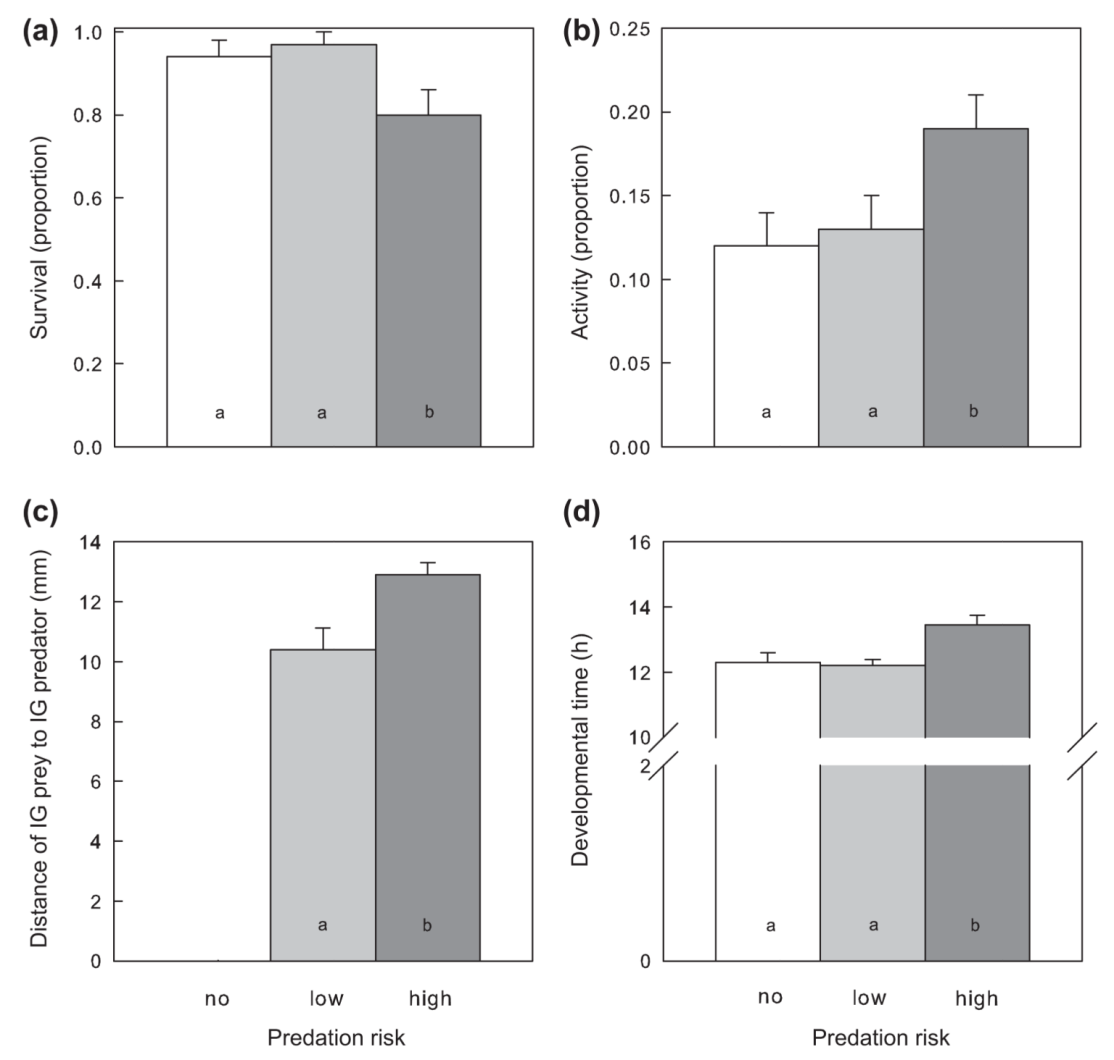
Influence of IGP risk (no: white, n = 39; low: light grey, n = 37; high: dark grey, n = 46) on survival (a), activity (b), spatial predator avoidance (c) and developmental time (d) of IG prey larvae of *P. persimilis* (mean + SE). Different letters inside bars indicate significant differences between predation risks (LSD-tests, p < 0.05).

Survival (GLM; Wald χ^2^_2_ = 7.570, p = 0.023), activity (χ^2^_2_ = 7.079, p = 0.029), spatial IG predator avoidance (χ^2^_1_ = 9.037, p = 0.003) and developmental times (χ^2^_2_ = 13.385, p = 0.001) of IG prey larvae were influenced by IGP risk. IG prey survival was lower in presence of the high risk IG predator than in the other treatments (Fig. 1a). Similarly, only presence of the high risk IG predator induced higher activity in IG prey (Fig. 1b) and increased the inter-individual distance between IG prey and IG predator (Fig. 1c). IG predator activity was not affected by IG predator species (mean proportion of time spent moving ± SE; *Neoseiulus californicus:* 0.32 ± 0.04; *Amblyseius andersoni*: 0.33 ± 0.04; GLM, Wald χ^2^_1_ = 0.022, p = 0.883). Only presence of the high risk IG predator prolonged the larval developmental time of IG prey as compared to the control (Fig. 1d). Larval IGP risk (GLM; Wald χ^2^_2_ = 0.339, p = 0.844) did not affect the sex ratio (female proportion) of IG prey reaching adulthood (no risk: 0.70; low IGP risk: 0.67; high IGP risk: 0.64), indicating that survival of IG prey was not sex-specific.

### Effects of larval IGP risk on nymphal development and behaviour

**Figure 2:**
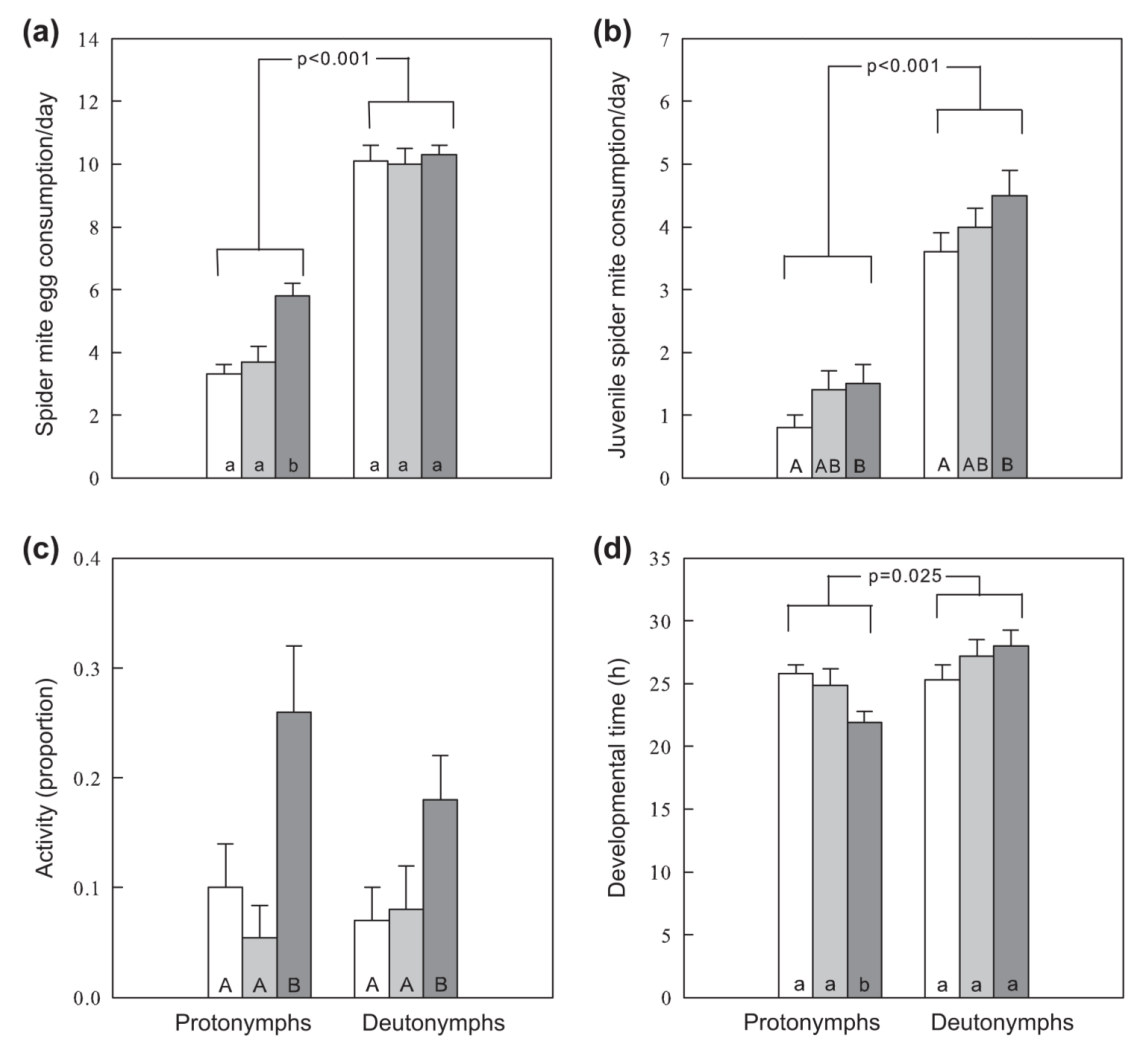
Influence of previous IGP risk during the larval stage (no: white, n = 36; low: light grey, n = 36); high: dark grey, n = 36) on consumption of spider mite eggs (a), consumption of spider mite juveniles (b), activity (c) and development (d) of *P. persimilis* protonymphs and deutonymphs (mean + SE). Different lower script and capital letters inside bars indicate significant effects between IGP risk levels within nymphal stages and between IGP risk levels pooled over nymphal stages, respectively (LSD-tests, p < 0.05). p-values refer to differences between proto- and deutonymphs pooled over IGP risk levels (GEE).

Spider mite egg consumption by nymphs was influenced by IGP risk of larvae (GEE, Wald χ^2^_2_ = 14.497, p = 0.001), nymphal stage (Wald χ^2^_1_ = 364.989, p < 0.001) and their interaction (Wald χ^2^_2_ = 6.927, p = 0.031). The significant interaction term indicated that egg consumption by deutonymphs was not affected by larval IGP risk, whereas protonymphs that had been exposed to high IGP risk in the larval stage consumed more spider mite eggs than protonymphs in the other treatments (Fig. 2a). Consumption of mobile juvenile spider mites by nymphs was affected by larval IGP risk (Wald χ^2^_2_ = 6.174, p = 0.046) and nymphal stage (Wald χ^2^_1_ = 123.135, p < 0.001) but not their interaction (Wald χ^2^_2_ = 1.004, p = 0.605). Pooled over nymphal stages, nymphs that had been exposed to high IGP risk in the larval stage consumed more juvenile spider mites (mean per day ± SE; 2.97 ± 0.21) than nymphs in the control treatment (2.21 ± 0.22). Pooled over larval IGP risks, deutonymphs consumed more juvenile spider mites (4.05 ± 0.17) than protonymphs (1.20 ± 0.18) (Fig. 2b).

The activity of nymphs was influenced by larval IGP risk (GEE; Wald χ^2^_2_ = 14.866, p = 0.001) but not nymphal stage (Wald χ^2^_1_ = 0.697, p = 0.404) and the interaction (Wald χ^2^_2_ = 1.899, p = 0.387). Nymphs that had been exposed to high IGP risk in the larval stage were more active (mean proportion ± SE; 0.21 ± 0.3) than nymphs in the other treatments (no IGP risk: 0.09 ± 0.03, low IGP risk: 0.07 ± 0.03) (Fig. 2c).

The developmental times of nymphs were affected by nymphal stage (GEE; Wald χ^2^_1_ = 4.993, p = 0.025) but not by larval IGP risk (Wald χ^2^_2_ = 3.660, p = 0.160). The effect of nymphal stage, however, depended on larval IGP risk (Wald χ^2^_2_ = 6.334, p = 0.042). While the developmental times of deutonymphs were not affected by larval IGP risk, protonymphs that had been exposed to high IGP risk in the larval stage developed more quickly than protonymphs in the other treatments (Fig. 2d).

### Effects of larval IGP risk on sex, age and size at maturity

**Figure 3:**
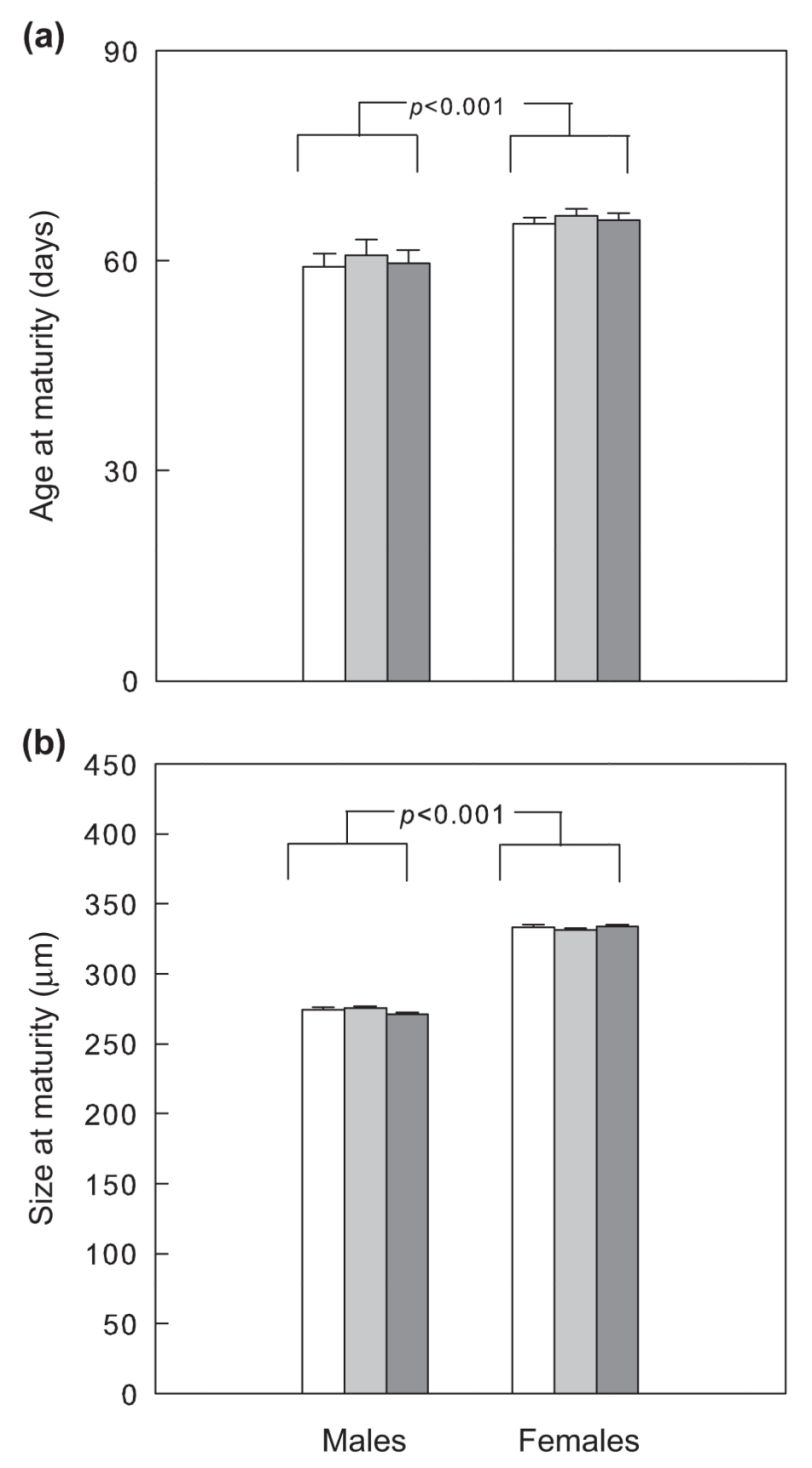
Influence of previous IGP risk during the larval stage (no: white, n = 36; low: light grey, n = 36); high: dark grey, n = 36) on age (a) and size (dorsal shield length) (b) at maturity of male and female *P. persimilis* (mean + SE). p-values refer to differences between males and females pooled over IGP risk levels (GLM).

Male *Phytoseiulus persimilis* developed more quickly from larva to adulthood than females (GLM; Wald χ^2^_1_ = 36.938, p < 0.001). The total developmental time from larva to adulthood was not influenced by larval IGP risk (Wald χ^2^_2_ = 1.500, p = 0.472) and the interaction between sex and larval IGP risk (Wald χ^2^_2_ = 0.389, p = 0.823) (Fig. 3a). Adult body size of males was smaller than that of females (Wald χ^2^_1_ = 1715.237, p < 0.001) but body size was not influenced by larval IGP risk (Wald χ^2^_2_ = 1.221, p = 0.543) and the sex/larval IGP risk interaction (Wald χ^2^_2_ = 1.786, p = 0.409) (Fig. 3b).

## Discussion

Our study indicates that the predatory mite *Phytoseiulus persimilis* is able to compensate for retarded development, induced by IGP risk early in life, by accelerating growth once conditions improved. These findings are consistent with compensatory but not catch-up growth (Hector and Nakagawa 2012). Our study represents the first experimental evidence that transient predation risk may similarly trigger compensatory growth in animals as unfavourable climatic conditions, food limitation or toxic substances (Dmitriew 2011).

### Proximate causes of compensatory growth

Predator presence usually reduces foraging activity and hence growth rates of prey (Werner and Anholt 1993, Gotthard 2000, Biro et al. 2004). Rare examples of inverse prey responses come from salamander and red frog larvae exposed to gape-limited predators. Prey increased their foraging activity and accelerated growth to reach earlier a body size freeing them from gape-limited predation (Vonesh and Bolker 2005, Urban 2007, 2008). In our experiments, prey increased its activity in the presence of the high-risk IG predator but this was not related to foraging because *P. persimilis* larvae do not feed (Schausberger and Croft 1999). Consequently, the physiological costs of increased activity of IG prey larvae in presence of the high-risk IG predator were not compensated for by increased food intake and hence prolonged their developmental times. Protonymphs that had been exposed to the high-risk IG predator in the larval stage were also more active than protonymphs in the other treatments. Contrary to larval activity, protonymphal activity should be mainly attributed to foraging but not anti-predator response to chemical IG predator traces because higher activity correlated positively with higher consumption rates. Moreover, we previously showed that chemical IG predator traces, which were still present after removal of the IG predators, only affect the walking path shape but not activity of IG prey (Walzer and Schausberger 2013a). As a consequence of higher consumption rates, protonymphs previously (in the larval stage) exposed to the high risk IG predator developed more quickly than those previously exposed to no or the low risk IG predator. Based on the generally acknowledged tradeoff between developmental time and body size (Stearns 1989, 1992, Roff 2001, Walzer and Schausberger 2011b for *P. persimilis*) longer developmental times of larvae decreased their growth rates but did not affect their size. We did not measure larval size here because we followed the same individuals throughout development, precluding stage-specific body size measurements requiring killing. However, it is true for phytoseiid mites in general that larval developmental time is highly plastic in response to environmental factors whereas larval size is robust and largely maternally determined (Croft et al. 1999). Moreover, from the larval and nymphal developmental times, nymphal consumption rates and size at maturity it is clear that protonymphs only compensated for retarded larval development whereas larval and nymphal size were unaffected by larval IGP risk. Protonymphs arising from larvae exposed to high IGP risk fed more and developed more quickly than protonymphs previously exposed to no or low IGP risk. Quicker development associated with higher consumption rates resulted in higher growth rates and excludes that these protonymphs simultaneously compensated for an alleged size deficit at the beginning of this stage (Stearns 1989, 1992, Roff 2001). Deutonymphal development and prey consumption and size at maturity were unaffected by larval IGP risk. Consequently, we argue that protonymphs balanced retarded larval development by compensatory but not catch-up growth, allowing reaching maturity at optimal age and size.

### Ultimate causes of compensatory growth

Whether reduced growth triggered by transient environmental stress may be later compensated or not depends, among other factors, on the type of stressor. For example, tadpoles were able to compensate for reduced body size development early in life via later catch-up growth when body size development was impaired by low temperatures but not food restriction (Dahl et al. 2012).The authors argued that food restriction is a rare stressor for tadpoles, thus not selecting for compensatory responses. In contrast, the stressor used in our experiments, IGP risk, is a strong selective force, which is evident in the evolution of complex interspecific threat-sensitive anti-predator behaviours of *P. persimilis*. These adaptations include abilities to predator species recognition and assessment of species-specific predation risk by both IG prey larvae and their mothers (Walzer and Schausberger 2011a, 2012, 2013a, b). IG prey larvae responded to predation risk in a threat-sensitive manner by increased activity and spatial avoidance of the high-risk predator at the expense of prolonged development. Age at maturity is a key life history trait in species with high intrinsic rates of increase (r_m_), such as *P. persimilis* (Sabelis 1985). Retarded development has negative fitness consequences for *P. persimilis*. Due to using an ephemeral prey resource, developmental time correlates with juvenile survival chance because prolonged developmental time increases the likelihood of food depletion and encounters with con- and heterospecific predators (Walzer and Schausberger 2011b). Thus, we argue that compensatory growth in *P. persimilis* after transient exposure to high IGP risk evolved to counter-balance the costs of anti-predator behaviour. Nonetheless, individual growth is considered to be evolutionarily optimized rather than maximized under ideal conditions (Arendt 1997, Dmitriew 2011) because developing too quickly can lower the cell functioning efficiency, immune function and resistance to physiological stressors (Mangel and Stamps 2001).The fitness benefits of compensatory growth are usually traded off against costs to be paid later in life (Metcalfe and Monaghan 2001), which need yet to be assessed for *P. persimilis*.

## Supplementary Material

Supplementary file
