# Supplementary material for: Compensatory growth following transient intraguild predation risk in predatory mites
Source: Oikos. Author manuscript; Available in PMC 2015 May 22. (PMC4441005; doi:10.1111/oik.01687)
Supplement: Supplementary file [file NIHMS63452-supplement-Supplementary_file.pdf]

Oikos

OIK-01687

Walzer, A., Lepp, N. and Schausberger, P. 2014. Compensatory growth following transient intraguild predation risk in predatory mites. – Oikos doi: 10.1111/oik.01687

Appendix 1

In a separate Excel-file (Data_file_a1) are data from the experiments presented in separate working sheets (WS) 1: Larval development and behaviour; WS 2: Nymphal development and behaviour; WS 3: age and size at maturity.

A	B	C	D	E	F	G	H	I
1	0	15	1	0		0		
2	0	12	1	0		0		
3	0	12	1	0		0		
4	0	12	1	0		0		
5	0	12	1	0		0		
6	0	12	1	0		0		
7	0	12	1	0.25		0.52		
8	0	12	1	0.25		0.52		
9	0	15	1	0		0		
10	0	10	1	0.25		0.52		
11	0	9	1	0		0		
12	0	12	1	0		0		
13	0	13	1	0		0		
14	0	12	1	0		0		
15	0	15	1	0		0		
16	0	12	1	0		0		
17	0		0					
18	0	12	1	0		0		
19	0	12	1	0.25		0.52		
20	0	9	1	0		0		
21	0	12	1	0		0		
22	0	12	1	0.25		0.52		
23	0	15	1	0.2		0.46		
24	0	15	1	0.25		0.52		
25	0	15	1	0		0		
26	0	12	1	0		0		
27	0		0					
28	0	12	1	0.25		0.52		
29	0	12	1	0.25		0.52		
30	0	12	1	0.5		0.79		
31	0	12	1	0.25		0.52		
32	0	12	1	0.25		0.52		
33	0	12	1	0.25		0.52		
34	0	12	1	0		0		
35	0	12	1	0		0		
36	0	15	1	0.25		0.52		
37	0	9	1	0.2		0.46		
38	0	9	1	0.2		0.46		
39	0	15	1	0.2		0.46		
1	1	12	1	0.25	0.5	0.52	0.79	6.75
2	1	15	1	0.4	0.4	0.68	0.68	10.8
3	1	12	1	0.25	0.25	0.52	0.52	12
4	1	15	1	0.4	0.4	0.68	0.68	9.6
5	1	12	1	0	0.5	0	0.79	11.5
6	1	15	1	0.4	0.4	0.68	0.68	9.6
7	1	12	1	0	0	0	0	10
8	1	12	1	0	0.25	0	0.52	9.75
9	1	12	1	0	1	0	1.57	10.25
10	1	12	1	0.25	0.25	0.52	0.52	15

Column A = replicates
Column B = treatment
0=control, 1=low risk, 2=high risk
Column C = larval development in days
Column D = survival, 0=dead, 1=alive
Column E = prey activity (proportion)
Column F = predator activity (proportion)
Column G = transformed prey activity
Column H= transformed predator activity
Column I= distance between prey and predator

11	1	12	1	0	0.25	0	0.52	16
12	1	12	1	0.25	0.5	0.52	0.79	6
13	1	15	1	0	0.4	0	0.68	12.6
14	1	15	1	0	0.25	0	0.52	12
15	1	12	1	0.25	0.25	0.52	0.52	7.5
16	1	12	1	0	0.25	0	0.52	5.5
17	1	12	1	0.25	0.25	0.52	0.52	14
18	1	15	1	0	0.4	0	0.68	17
19	1	12	1	0	0.25	0	0.52	9.75
20	1	12	1	0	0.25	0	0.52	4.75
21	1	12	1	0.25	0.5	0.52	0.79	8.5
22	1	15	1	0.2	0.4	0.46	0.68	14.2
23	1	12	1	0	0.25	0	0.52	6.5
24	1	12	1	0.25	0.5	0.52	0.79	3.25
25	1	12	1	0.2	0	0.46	0	15.25
26	1	15	1	0	0	0	0	15.2
27	1	15	1	0.25	0.2	0.52	0.46	16
28	1	12	1	0	0.5	0	0.79	9.25
29	1	12	1	0	0.25	0	0.52	5.5
30	1	12	1	0.25	0	0.52	0	11.5
31	1	15	1	0.25	0.5	0.52	0.79	12
32	1	12	1	0	0.25	0	0.52	4.2
33	1	12	1	0	0.5	0	0.79	8
34	1	12	1	0.25	0	0.52	0	11.5
35	1	12	0	0.25	0	0.52	0	6.25
36	1	9	1	0.25	0	0.52	0	25
37	1	9	1	0	1	0	1.57	18.33
1	2		0					
2	2	12	1	0.25	0.25	0.52	0.52	11
3	2	15	1	0.2	0.4	0.46	0.68	11.8
4	2	15	1	0.2	0.4	0.46	0.68	13.4
5	2		0					9
6	2	15	1	0.4	0	0.68	0	13
8	2	15	1	0.2	0.6	0.46	0.89	12
9	2		0					
10	2	12	1	0.25	0.25	0.52	0.52	11.25
11	2	15	1	0.2	0	0.46	0	17
12	2	15	1	0.2	0.2	0.46	0.46	14
13	2	15	1	0.2	0.2	0.46	0.46	11.6
14	2		0					8
15	2	12	1	0.2	0.5	0.46	0.79	9
16	2	15	1	0.2	0.2	0.46	0.46	19.6
17	2	15	1	0.2	1	0.46	1.57	9.6
18	2	15	1	0	0.4	0	0.68	8.8
19	2	12	1	0.25	0.5	0.52	0.79	9.25
20	2	15	1	0	0.2	0	0.46	18.8
21	2		0					19
22	2		0					13
23	2	15	1	0.25	0.75	0.52	1.05	16.25
24	2	12	1	0	0	0	0	13

25	2	12	1	0	0.4	0	0.68	11.8
26	2	12	1	0	0.25	0	0.52	12
28	2		0					10.5
29	2	12	1	0	0.25	0	0.52	15
30	2	12	1	0.4	0.6	0.68	0.89	9.8
31	2	15	1	0.25	0.25	0.52	0.52	15
32	2	15	0					12
33	2	15	1	0.25	0.5	0.52	0.79	14.25
34	2	12	1	0	0.75	0	1.05	13.25
35	2	18	1	0.25	0.5	0.52	0.79	13.25
36	2		0					9.33
37	2		0					17
38	2	12	1	0.4	0.2	0.68	0.46	17.4
39	2	12	1	0.2	0.2	0.46	0.46	15.2
40	2	12	1	0.25	0	0.52	0	12.5
41	2	15	1	0	0	0	0	13.25
42	2	12	1	0.32	0.33	0.6	0.61	17.5
43	2	12	1	0.25	0.5	0.52	0.79	13
44	2	12	1	0	0.25	0	0.52	10.75
45	2	12	1	0.25	0	0.52	0	13.5
46	2	12	1	0.5	0.5	0.79	0.79	10
47	2	12	1	0.2	0.2	0.46	0.46	10.6
48	2	12	1	0.25	0.25	0.52	0.52	11.5

A	B	C	D	E	F	G	H
0	1	1	24.5	1	4	0	1.57
0	2	0	18.5	1	1	2	0
0	3	0	30.5	1	6	4	0
0	4	0	30.5	1	7	4	0
0	5	0	27.5	1	3	2	0
0	6	0	18.5	1	2	0	0
0	7	0	18.5	1	1	0	0
0	8	0	18.5	1	3	0	0
0	9	0.5	27.5	1	2	1	0.79
0	10	0	24.5	1	3	0	0
0	11	0	18.5	1	2	0	0
0	12	0	30.5	1	8	0	0
0	13	0	18.5	1	3	0	0
0	14	50	18.5	1	2	0	0.79
0	15	0	24.5	1	2	0	0
0	16	0.5	27.5	1	2	2	0.79
0	18	0	24.5	1	2	3	0
0	19	0.5	30.5	1	4	4	0.79
0	20	0	30.5	1	4	0	0
0	21	0	30.5	1	5	1	0
0	22	0	27.5	1	5	3	0
0	23	0	27.5	1	4	0	0
0	24	0.5	24.5	1	2	0	0.79
0	25	0	27.5	1	3	0	0
0	26	0	30.5	1	2	0	0
0	28	0	27.5	1	3	0	0
0	29	0	27.5	1	2	2	0
0	30	0	27.5	1	3	1	0
0	31	0	27.5	1	2	3	0
0	32	0	27.5	1	2	0	0
0	33	0	27.5	1	3	0	0
0	34	0.5	27.5	1	3	0	0.79
0	35	0	30.5	1	2	0	0
0	36	0	24.5	1	5	0	0
0	37	0	24.5	1	2	0	0
0	38	0	24.5	1	2	0	0
0	1	0.5	24	2	6	3	0.79
0	2	0	24	2	6	7	0
0	3	0	24	2	7	4	0
0	4	0	24	2	9	3	0
0	5	0	12	2	7	2	0
0	6	0	36	2	9	4	0
0	7	0	24	2	8	2	0
0	8	0	36	2	7	1	0
0	9	0	12	2	7	1	0
0	10	0	36	2	9	2	0
0	11	0.33	36	2	11	1	0.61
0	12	0	24	2	8	4	0
0	13	0	36	2	11	3	0

Column A: treatment
control=0, low risk=1, high risk=2
Column B: replicate
Column C: activity (proportion)
Column D: development in h
Column E: developmental stage
protonymph=1
deutonymph)=2
Column F: consumed spider mite eggs/day
Column G: consumed mobile spider mites per day
Column H: transformed activity

0	14	0	36	2	14	6	0
0	15	0.33	12	2	7	6	0.61
0	16	0	24	2	6	5	0
0	18	0	24	2	8	8	0
0	19	0	24	2	7	1	0
0	20	0	24	2	10	3	0
0	21	0	24	2	13	2	0
0	22	0	24	2	8	2	0
0	23	0	24	2	9	5	0
0	24	0.5	24	2	10	6	0.79
0	25	0	24	2	10	5	0
0	26	0	24	2	7	4	0
0	28	0.5	24	2	12	4	0.79
0	29	0	24	2	13	3	0
0	30	0	12	2	14	3	0
0	31	0	24	2	10	8	0
0	32	0	24	2	14	5	0
0	33	0	24	2	14	3	0
0	34	0	24	2	8	2	0
0	35		36	2	18	7	
0	36	0	24	2	12	7	0
0	37	0.33	36	2	11	4	0.61
0	38	0	24	2	13	3	0
1	1	0	24.5	1	2	4	0
1	2	0	27.5	1	7	6	0
1	3	0	18.5	1	4	0	0
1	4	0	27.5	1	5	3	0
1	5	0	30.5	1	7	2	0
1	6	0	27.5	1	5	3	0
1	7	0	27.5	1	6	3	0
1	8	0	30.5	1	4	1	0
1	9	0	24.5	1	5	0	0
1	10	0	24.5	1	4	0	0
1	11	0	18.5	1	2	0	0
1	12	0	18.5	1	3	0	0
1	13	0	27.5	1	4	0	0
1	14	0	18.5	1	2	0	0
1	15	0	18.5	1	1	1	0
1	16	0	27.5	1	2	2	0
1	17	0	27.5	1	2	0	0
1	18	0	27.5	1	4	2	0
1	19	0	27.5	1	2	3	0
1	20	0	27.5	1	3	4	0
1	21	0	27.5	1	3	0	0
1	22	0	24.5	1	3	1	0
1	23	0.5	27.5	1	2	4	0.79
1	24	0	30.5	1	2	0	0
1	25	0	18.5	1	4	0	0
1	26	0	24.5	1	5	1	0
1	27	0	27.5	1	4	0	0

1	28	0	27.5	1	2	2	0
1	29	0	27.5	1	2	4	0
1	30	0.5	27.5	1	6	0	0.79
1	31	0	24.5	1	4	0	0
1	32	0	27.5	1	3	0	0
1	33	0.33	27.5	1	2	0	0.61
1	34	0.5	27.5	1	4	3	0.79
1	36	0	27.5	1	9	0	0
1	37	0.5	24.5	1	4	2	0.79
1	1	0	24	2	7	2	0
1	2	0	24	2	8	6	0
1	3	0	24	2	11	2	0
1	4	0	24	2	7	7	0
1	5	0	24	2	10	7	0
1	6	0	24	2	10	7	0
1	7	0	24	2	13	4	0
1	8	1	24	2	8	7	1.57
1	9	0.33	36	2	9	0	0.61
1	10	0.33	36	2	10	1	0.61
1	11	0.33	36	2	9	5	0.61
1	12	0	36	2	7	3	0
1	13	0	24	2	10	4	0
1	14	0	36	2	9	4	0
1	15	0	36	2	10	3	0
1	16	0	24	2	8	5	0
1	17	0	24	2	8	6	0
1	18	0	24	2	9	3	0
1	19	0.5	24	2	11	3	0.79
1	20	0	12	2	8	4	0
1	21	0	12	2	9	2	0
1	22	0	24	2	8	2	0
1	23	0	36	2	10	4	0
1	24	0	12	2	13	7	0
1	25	0	36	2	11	4	0
1	26	0.33	36	2	10	4	0.61
1	27	0	24	2	12	5	0
1	28	0.5	36	2	9	5	0.79
1	29	0	24	2	5	2	0
1	30	0	24	2	9	5	0
1	31	0	36	2	16	4	0
1	32	0	24	2	12	2	0
1	33	0	36	2	18	2	0
1	34	0	24	2	16	4	0
1	36	0	24	2	9	6	0
1	37	0	24	2	6	3	0
2	2	0.5	27.5	1	12	4	0.79
2	3	0.5	24.5	1	6	2	0.79
2	4	0	15.5	1	9	0	0
2	6	0.5	27.5	1	8	2	0.79
2	8	0.5	27.5	1	8	5	0.79

2	10	0.5	18.5	1	7	2	0.79
2	11	0	15.5	1	4	2	0
2	12	0.5	18.5	1	4	5	0.79
2	13	0	24.5	1	7	5	0
2	15	0	27.5	1	5	5	0
2	16	0	15.5	1	9	2	0
2	17	0	15.5	1	3	5	0
2	18	0	15.5	1	3	1	0
2	19	0.5	27.5	1	4	0	0.79
2	20	0.5	27.5	1	6	0	0.79
2	23	0	15.5	1	2	2	0
2	24	0	18.5	1	4	0	0
2	25	1	15.5	1	2	0	1.57
2	26	0	18.5	1	9	3	0
2	27	0	15.5	1	3	0	0
2	29	1	18.5	1	5	0	1.57
2	30	0.5	15.5	1	3	0	0.79
2	31	0	27.5	1	5	0	0
2	32	0.5	18.5	1	5	3	0.79
2	33	0.5	24.5	1	2	0	0.79
2	34	0	27.5	1	6	6	0
2	35	1	18.5	1	5	0	1.57
2	38	0	27.5	1	9	0	0
2	39	0	27.5	1	6	3	0
2	40	0	27.5	1	6	3	0
2	41	0	27.5	1	8	0	0
2	42	0	27.5	1	5	0	0
2	43	0	27.5	1	5	3	0
2	44	1	18.5	1	5	0	1.57
2	46	0.5	24.5	1	3	2	0.79
2	47	0	27.5	1	5	5	0
2	2	0	24	2	11	3	0
2	3	1	24	2	12	3	1.57
2	4	0	36	2	12	8	0
2	6	0	12	2	10	6	0
2	8	0	36	2	9	8	0
2	10	0	36	2	8	3	0
2	11	0	24	2	4	3	0
2	12	0.33	24	2	4	3	0.61
2	13	0	24	2	10	4	0
2	15	0.5	24	2	10	5	0.79
2	16	0	36	2	12	6	0
2	17	0.33	24	2	10	3	0.61
2	18	0.33	36	2	9	4	0.61
2	19	0	24	2	9	2	0
2	20	0.5	24	2	12	2	0.79
2	23	0.5	24	2	8	2	0.79
2	24	0	36	2	11	7	0
2	25	0.5	24	2	12	3	0.79
2	26	0.33	36	2	12	6	0.61

2	27	0.33	36	2	7	3	0.61
2	29	0.33	36	2	11	6	0.61
2	30	0	36	2	12	8	0
2	31	0.5	24	2	13	3	0.79
2	32	0	24	2	12	6	0
2	33	0	12	2	11	3	0
2	34	0	24	2	14	4	0
2	35	0	36	2	8	5	0
2	38	0	24	2	12	1	0
2	39	0.33	36	2	8	1	0.61
2	40	0	12	2	10	4	0
2	41	0	24	2	11	8	0
2	42	0	24	2	13	5	0
2	43	0.5	24	2	12	6	0.79
2	44	0	36	2	11	3	0
2	46	0.5	24	2	14	4	0.79
2	47	0	36	2	8	5	0

A	B	C	D	E
control	1	female	329	63.5
control	2	female	329	54.5
control	3	female	326	66.5
control	4	female	322	66.5
control	5	male	271	51.5
control	6	male	283	66.5
control	7	male	281	54.5
control	8	female	338	66.5
control	9	male	266	54.5
control	10	female	342	72.5
control	11	male	276	63.5
control	12	female	323	66.5
control	13	female	338	66.5
control	14	female	340	66.5
control	15	male	285	51.5
control	16	female	332	63.5
control	18	female	334	66.5
control	19	female	336	66.5
control	20	female	345	63.5
control	21	female	334	66.5
control	22	female	327	63.5
control	23	female	335	66.5
control	24	female	324	63.5
control	25	male	276	66.5
control	26	female	333	66.5
control	28	female	328	63.5
control	29	female	340	63.5
control	30	male	277	51.5
control	31	male	265	63.5
control	32	female	331	63.5
control	33	female	346	63.5
control	34	male	271	63.5
control	35	female	345	78.5
control	36	female	332	63.5
control	37	female	330	69.5
control	38	female	330	57.5
ɔw risk predatc	1	male	276	60.5
ɔw risk predatc	2	female	341	66.5
ɔw risk predatc	3	female	339	54.5
ɔw risk predatc	4	female	321	66.5
ɔw risk predatc	5	female	332	66.5
ɔw risk predatc	6	female	328	66.5
ɔw risk predatc	7	female	335	63.5
ɔw risk predatc	8	female	333	66.5
ɔw risk predatc	9	female	325	66.5
ɔw risk predatc	10	female	320	66.5
ɔw risk predatc	11	female	338	66.5
ɔw risk predatc	12	male	272	63.5
ɔw risk predatc	13	female	331	66.5

Column A: treatment (no, low, high IGP risk)
Column B: replicate
Column C: sex
Column D: dorsal shield length (microns)
Column E: development from larva to adulthood in h

ɔw risk predatc	14	female	334	69.5
ɔw risk predatc	15	female	334	66.5
ɔw risk predatc	16	male	276	63.5
ɔw risk predatc	17	female	331	63.5
ɔw risk predatc	18	female	324	66.5
ɔw risk predatc	19	female	321	63.5
ɔw risk predatc	20	male	279	51.5
ɔw risk predatc	21	male	269	51.5
ɔw risk predatc	22	male	271	63.5
ɔw risk predatc	23	female	314	75.5
ɔw risk predatc	24	male	278	54.5
ɔw risk predatc	25	female	335	66.5
ɔw risk predatc	26	female	323	75.5
ɔw risk predatc	27	female	347	66.5
ɔw risk predatc	28	female	338	66.6
ɔw risk predatc	29	male	281	63.5
ɔw risk predatc	30	female	335	63.5
ɔw risk predatc	31	male	274	75.5
ɔw risk predatc	32	female	338	63.5
ɔw risk predatc	33	female	340	75.5
ɔw risk predatc	34	female	332	63.5
ɔw risk predatc	36	male	273	60.5
ɔw risk predatc	37	male	273	60.5
gh risk predat	2	female	330	63.5
gh risk predat	3	male	285	63.5
gh risk predat	4	female	322	66.5
gh risk predat	6	female	325	54.5
gh risk predat	8	female	331	78.5
gh risk predat	10	female	330	66.5
gh risk predat	11	male	270	54.5
gh risk predat	12	female	333	63.5
gh risk predat	13	female	337	63.5
gh risk predat	15	female	333	63.5
gh risk predat	16	female	341	66.5
gh risk predat	17	male	270	54.5
gh risk predat	18	female	333	66.5
gh risk predat	19	male	274	63.5
gh risk predat	20	female	318	66.5
gh risk predat	23	male	268	54.5
gh risk predat	24	male	273	66.5
gh risk predat	25	male	271	51.5
gh risk predat	26	female	331	66.5
gh risk predat	27	male	265	66.5
gh risk predat	29	female	326	66.5
gh risk predat	30	female	345	63.5
gh risk predat	31	female	335	66.5
gh risk predat	32	female	349	66.5
gh risk predat	33	male	266	51.5
gh risk predat	34	male	273	63.5
gh risk predat	35	male	276	72.5

gh risk predat	38	female	321	63.5
gh risk predat	39	female	342	75.5
gh risk predat	40	male	268	51.5
gh risk predat	41	female	340	66.5
gh risk predat	42	female	334	63.5
gh risk predat	43	female	321	63.5
gh risk predat	44	female	342	75.5
gh risk predat	46	male	273	51.5
gh risk predat	47	female	340	66.5
